# The exit from naive pluripotency: a platform for the study of enhancer mechanistics

**DOI:** 10.1042/BST20253037

**Published:** 2025-08-18

**Authors:** Mattias Enar Jonasson, Christa Buecker

**Affiliations:** 1 Max Perutz Labs, University of Vienna, Vienna Biocenter Campus (VBC), Vienna 1030, Austria; 2 Vienna Biocenter PhD Program, Doctoral School of the University of Vienna and the Medical University of Vienna, Vienna 1030, Austria

**Keywords:** cell state transition, cis-regulatory elements, enhancer, exit of naive pluripotency, transcriptional regulation

## Abstract

Multicellular life depends on the ability to activate and repress genes in a highly context-specific manner. With each cell state transition, a new transcriptional profile is established. As non-coding DNA elements, enhancers mediate their regulatory potential through the effectors they recruit. While ultimately instructed by the underlying DNA sequence, enhancer activity depends on several factors, such as transcription factor availability, chromatin state, and promoter proximity, all of which are dynamically regulated within the cell. Even when we understand the regulation of one enhancer, its genomic impact is dependent on its integration within the regulatory landscape. Thus, a full picture of enhancer dynamics can only be painted through broad, but controlled, approaches that integrate investigations into multiple levels of gene regulatory mechanisms. In this review, we will present the exit of naive pluripotency as a prime setting to do just that and contextualize how its contemporary use has been, and could be, used to reveal the intricacies of enhancer mechanistics.

## Introduction

Spatiotemporal control of gene expression is central to the development of complex organisms, and the mechanisms that allow it remain a source of intrigue within modern biology. Promoters are limited in how much information can be encoded to instruct when and where the associated gene should be expressed. Consequently, the origin of multicellular life might have been possible through the evolution of additional gene regulatory elements [1]. Genetic innovation gave rise to regulatory elements that could act upon target genes from a distance, complementing the local mode of gene regulation that is presently associated with the transcription of housekeeping genes [2]. A broadened regulatory landscape meant that gene expression could be adapted to increasingly specific stimuli, allowing for complex concerted efforts between cells. Development is an ancient testament to the success of distal cis-regulatory elements, but the mechanistics by which they drive it are still being unraveled.

Central among cis-regulatory elements are enhancers—non-coding DNA sequences that support distal promoters in tuning the transcriptional response to cellular needs. Active enhancers recruit context-specific cofactors that ultimately act upon the associated gene promoters to promote their appropriate regulation in a poorly understood fashion. Recruiting transcriptional regulators in a sequence-specific manner, enhancers provide a physical extension to the regulatory environment of genes, increasing both the flexibility and specificity with which promoters drive transcription. Enhancers have evolved to control their target genes in specific tissues and at specific times. Hence, they stand as key regulators of development.

Different combinations of enhancers are active in different cell types and states. Most genes are regulated by several enhancers, and their cis-regulatory interactions are important for developmental processes [3]. In humans, the median number of enhancers associated with a promoter is proposed to be three per gene [4]. Enhancer collaboration is emerging as a cornerstone of complex gene regulation. Despite being central drivers of development, enhancers are remarkably redundant, with the deletion of a single enhancer often having little-to-no effect on the outcome of mammalian development [5]. This phenomenon aligns with early discoveries in *Drosophila*, where the concept of ‘shadow enhancers’ was established and a network of functionally redundant enhancers that help ensure robustness in gene expression patterns was revealed [6–8].

Enhancers that exhibit astounding evolutionary conservation can also demonstrate redundancy [9]. In some cases, the deletion of an ultraconserved enhancer does cause deleterious, tissue-specific, developmental phenotypes [10], but it can also have paradoxically systemic effects rather than the expected organ-specific ones [11]. It is clear that the importance of enhancer collaboration stretches further than providing redundancy to deleterious mutations [12], but a lot is still left to learn about how enhancer-enhancer interactions promote precise gene expression.

How enhancers turn on and off, and how their subsequent activity affects gene expression, is not well understood. Multiple layers of regulatory mechanisms are integrated to decide enhancer activity, and while reductionist approaches start to tease apart their individual importance, enhancers can only be truly understood within their endogenous chromatin context. During development, cells traverse between sequential cell states, each characterized by a cell state-specific enhancer landscape. To establish novel cell types, these landscapes must be rewired. As such, cell state transitions are exemplary settings in which to study enhancer mechanistics. In the embryo, many transitions occur in parallel, resulting in a heterogeneous and complex environment that is difficult to probe using currently available experimental techniques. Studying a single cell state transition *in vitro* is, therefore, an excellent experimental system for studying the interplay of the different levels of enhancer regulation.

## The exit from naive pluripotency

**Figure 1:**
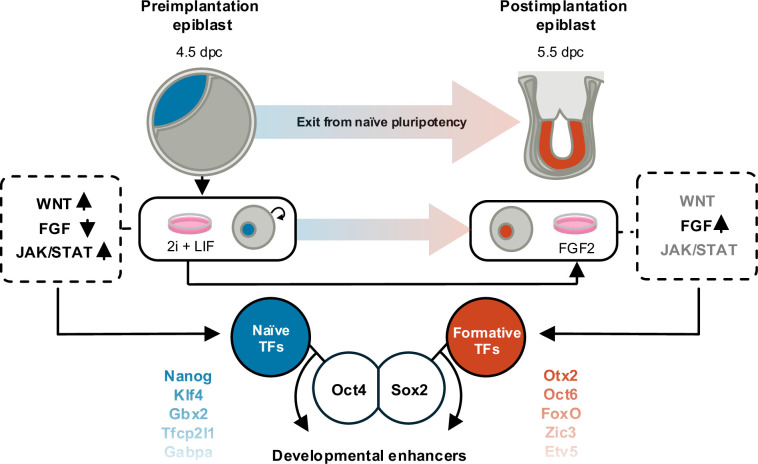
The exit from naive pluripotency. Mouse embryonic stem cells are derived from early embryos and resemble pre-implantation epiblast cells when cultured with two small-molecule inhibitors (2i) and LIF. 2i + LIF maintains a balance between key signaling pathways that promote a transcriptional state where specific transcription factors (TFs), such as NANOG and KLF4, work together with the core pluripotency components OCT4 and SOX2 to maintain naive pluripotency; a cell state of indefinite self-renewal. Upon removal of 2i + LIF, and with the addition of FGF2, naive cells are ‘released’ to differentiate in a process mimicking that of post-implantation epiblast cells, where the first step is called formative pluripotency. Here, the cells attain the ability to differentiate into specific lineages, with the interaction partners of the core components changing accordingly. The factors converge on developmental enhancers, bringing forth a major cell state transition through a myriad of molecular mechanisms. LIF, leukemia inhibitory factor, FGF2, Fibroblast Growth Factor 2

The exit from naive pluripotency is a developmentally important cell state transition that can easily be modeled through an efficient and tractable *in vitro* differentiation system (Figure 1). Mouse embryonic stem cells (mESCs) are capable of self-renewal while retaining their capacity to contribute to all tissues of the embryo proper. This is often referred to as naive pluripotency. *In vivo*, naive pluripotency is highly transient and attained only by cells in the pre-implantation epiblast [13]. Fortunately, two small-molecule inhibitors (2i) enforcing activation of Wnt-signaling and inhibition of FGF-signaling, together with leukemia inhibitory factor (LIF), are sufficient to capture and maintain the naive state indefinitely in *in vitro* cell culture. Despite being noncommitted, naive pluripotent cells have mounted the molecular machinery needed to exit pluripotency [14,15]. Through removal of 2i/LIF, cells can be released along the path of differentiation, establishing a new cellular identity resembling the epiblast of the early post-implantation embryo at E5.5.

The exit from naive pluripotency is fast, highly reproducible, and irreversible. After 2i/LIF withdrawal, all cells within the starting population exit naive pluripotency within 24–48 hours. However, not all cells initiate differentiation simultaneously. Perhaps surprisingly, the cell cycle is not the major source for the heterochronic exit from naive pluripotency [16–19]. The observed heterogeneity could instead arise from factors such as variations in the cellular microenvironment [20,21], metabolic cellular state [22], stochastic gene expression [23], or differences in the initial epigenetic landscape of the cells [24]. Despite being metachronous [17], cells exiting naive pluripotency follow a unidirectional differentiation trajectory [16,25,26]. In other words, the differentiation protocol is highly reproducible and irreversible.

As naive exit is deterministic, the system allows us to repeatedly probe different individual factors involved in cell state transitions. Over time, a holistic understanding of how they interact will emerge from parallel findings. The study of enhancer mechanistics during development relies on the integration of several layers of regulation; therefore, the exit from naive pluripotency is an excellent model system for dissection of the contribution of individual factors to enhancer activity. In this review, we will briefly summarize our current understanding of how different gene regulatory mechanisms determine enhancer activity and how they act within the context of the exit of naive pluripotency.

## Using naive pluripotency to study and contextualize enhancer mechanistics

Enhancer activity is orchestrated through multiple levels of regulation, each contributing to the precise control of gene expression. This complex interplay allows cells to dynamically respond to developmental cues and to transition between cellular states in the correct space and time. In this section, we examine the main regulatory layers that modulate enhancer function and explore how these interactions are essential for cell state transition by examining their role in the exit of naive pluripotency.

### Transcription factors

Transcription factors (TFs) are key regulators of cell state and enhancer activity. While the direct molecular action of TFs is often not clear, they are known to promote or repress transcription either directly or through recruiting effector proteins. TFs possess DNA-binding domains through which they recognize and bind to specific DNA sequence motifs. Enhancers are composed of clusters of motifs and, akin to genetic billboards, can promote the local attraction of TFs. In turn, the recruited factors act synergistically to promote the assembly of productive transcriptional complexes on promoters associated with the enhancer. Consequently, our understanding of enhancer mechanistics centers on the dynamics by which TFs interact with genomic DNA.

During naive pluripotency, the expression of specific pluripotency-related TFs maintains a gene regulatory network centered on developmental potency and self-renewal. TFs OCT4 and SOX2 form the core of this network [27], and together, they bind and activate most enhancers specific to pluripotency [28]. The core factors remain important throughout pre- and post-implantation development, but a plethora of TFs co-ordinate naive exit [15,27].

Interactions between TFs can influence their function, which includes what genes they target. Depending on the presence of accessory TFs, the binding patterns of the core factors and the effects of their associated enhancers change drastically. An example of this is OTX2, which is engaged in the early stages of naive exit [29]. It acts as an interaction partner to OCT4 to redirect its binding from naive pluripotency enhancers to those specific to early differentiation [28], thereby rewiring the enhancer landscape. Throughout naive exit, the expression of TFs that support the naive pluripotency network, such as KLF2 and TFCP2L1 [30,31], declines as signaling pathways are no longer active. Notably, some TFs specific to pluripotency, such as ESRRB, have been suggested to guide the core factors to new binding sites during the initial phase of differentiation [25]. Redirection of binding is one of several mechanisms through which TFs dynamically reshape enhancer activity during cell state transitions.

TFs can have direct effects on enhancer activity. In the exit of naive pluripotency, some TFs, such as FOXD3 [32], OCT6 [33], and ZIC3 [34], repress naive-specific enhancers and help dismantle the pluripotent state. Conversely, TFs can ensure that genes remain active even as their regulatory input changes. For example, GRHL2 defines which newly active enhancers sustain the expression of an already active gene [35]. Additionally, the expression of new TFs during this period can establish novel enhancer-promoter interactions, further promoting lineage commitment [36]. Thus, TFs not only determine which genes are expressed but also actively reshape the enhancer landscape, either by silencing regulatory elements tied to a previous state or by stabilizing new enhancer-promoter interactions that support differentiation.

While changes in TF expression levels can enforce lasting alterations in cell identity, cell state transitions are often driven by modulating the activity of pre-existing TFs. This can occur through cellular localization, as is the case of TFE3, which translocates out of the nucleus to allow the exit from naive pluripotency [37]. Similarly, nuclear export of KLF4, a TF that maintains long-range interactions at the *Oct4/Pou5F1* locus needed for pluripotency [38], triggers the exit of naive pluripotency [39]. Post-translational modifications also play a crucial role in regulating TF activity. OCT4 itself is the target of numerous post-translational modifications such as sumoylation [40], phosphorylation [41], and O-GlcnAc [42] that affect its stability, target binding, and transcriptional output. Ultimately, a TF’s ability to influence the genome depends not just on its presence or activity but also on whether it can access its target sites, a constraint that is itself tightly regulated at multiple levels.

### Chromatin accessibility

Chromatin refers to the dynamic composition of DNA and histone proteins, and it plays an essential role in how enhancers can be activated. To organize the DNA within the cell, DNA is wrapped around octamers of histone proteins forming nucleosomes. Organized DNA is known as chromatin, and at a higher level of organization, nucleosomes flexibly organize into a polymer melt-like structure with different levels of local compaction [43]. Open chromatin permits transcription factor binding to DNA and enables transcription, while closed chromatin restricts access and activity. However, accessibility is not binary; some factors can engage with compacted chromatin by specifically recognizing nucleosome-associated DNA or through recruiting chromatin remodelers. As a result, chromatin organization is not just a passive feature but actively shapes transcription by influencing which factors can bind in a given context. 

Active enhancers are typically associated with open chromatin; however, the mere presence of accessible chromatin does not necessarily correlate with enhancer activity. Enhancer activity is shaped by both chromatin accessibility and the transcriptional landscape. Some enhancers require specific chromatin environments to become active (‘chromatin-dependent enhancers’) [44], while others function within closed chromatin, remaining dormant until activated by environmental or developmental cues [45]. Notably, many open chromatin regions lack enhancer function altogether. Even within ‘super-enhancers’, not all constituent elements directly contribute to transcription. Instead, non-canonical regulatory elements act as ‘facilitators’, ensuring full enhancer function by enabling long-range regulatory interactions [46]. This is particularly relevant during development and cell state transitions, where dynamic changes in chromatin accessibility and transcription factor availability dictate which enhancers become active, ensuring precise spatiotemporal regulation of gene expression necessary for lineage commitment and differentiation.

Pluripotency is linked to a uniquely accessible and dynamic chromatin landscape. Naive pluripotent cells show lower levels of chromatin compaction [47] and greater diversity in nuclear condensate behavior [48] compared with differentiated cells. Notably, RNA polymerase II has been shown to form transient condensates that correlate with transcriptional bursting activity in embryonic stem cells [49]. During naive exit, these condensates disperse, coinciding with broader nuclear reorganization and enhancer inactivation [50]. Differentiation is marked by a progressive loss of chromatin accessibility at pluripotency genes and their enhancers [51]. In pluripotency, chromatin state is linked to the activity of pluripotency factors, with OCT4 depletion leading to chromatin condensation in epiblast cells [52]. OCT4 works together with chromatin remodelers such as BAF both in naive pluripotency and during its exit [28,53,54]. These interactions suggest that chromatin remodelers are recruited by the core pluripotency factors independent of their specific binding patterns.

High chromatin accessibility is critical for pluripotency, but certain regulatory elements require targeted repression to prevent uncontrolled activation. Highly compacted DNA, marked by the epigenetic histone modification trimethylation of lysine 9 of histone 3 (H3K9me3), restricts aberrant transcriptional programs and safeguards naive pluripotency from activation of transposable elements and cryptic enhancers [55]. Recent findings suggest that H3K9me3 is not uniformly stable across the genome but is maintained through distinct, region-specific dynamics, with some regions losing H3K9me3 rapidly upon perturbation, while others exhibit more stable repression, revealing a nuanced balance between accessibility and long-term silencing [56]. This highlights how chromatin accessibility is highly dynamic and tightly intertwined with different histone modifications.

### Histone modifications

In addition to chromatin remodelers, chromatin modifiers are recruited to enhancers to modify their structure and function, often through establishing post-translational modifications on histones. Numerous amino acid residues within the tail ends of histones are commonly modified, but the contribution of individual modifications to enhancer regulation remains unclear. Specific histone modifications correlate with different states of enhancer activity (see Table 1for a summary) and are often erroneously equated as causative [68]. Among the modifications, acetylation and methylation of lysines have received the most attention to date.

**Table 1:** Key epigenetic modifications commonly found on enhancers and their role in pluripotency.

Modification	Type	Writers	Erasers	Role in mESCs	Role in differentiation	Phenotype when disrupted	Citations
H3K4me1	Active	KMT2C/D (MLL3/4)	KDM1A (LSD1)	✔️ Required for enhancer priming	✔️ Required for lineage specification	Impaired activation of developmental enhancers	[57,58]
H3K27ac	Active	CBP, p300	HDAC1/2/3	❌ Not necessary	❌ Not necessary	Not essential; presence increases enhancer activity	[59,60]
H3K27me3	Repressive	PRC2: EZH2, EED, SUZ12	KDM6A/B (UTX/JMJD3)	❌ Not needed in steady state	✔️ Important for differentiation	Premature differentiation or failure of lineage commitment	[61–63]
H3K9me3	Repressive	KMT1A/B, KMT1E (SUV39H1/2, SETDB1)	KDM4A/B/C (JMJD2 family)	✔️ Required to silence aberrant transcription	✔️ Required for lineage commitment	De-repression of transposons and cryptic enhancers	[55,56,64]
H2AK119ub	Repressive	PRC1: RING1A/B	BAP1	❓ Context-dependent	✔️ Required for differentiation	Disrupted differentiation cues	[65,66]
DNA methylation (5mC)	Repressive	DNMT1, DNMT3A/B	TET1/2/3	✔️ Helps silence transposons	✔️ Important for lineage specification	Impaired lineage commitment	[67]

H3K9me3, trimethylation of lysine 9 of histone 3. mESC, mouse embryonic stem cell. PRC1, Polycomb-repressive complex 1. PRC2, Polycomb-repressive complex 2.

Histone acetylation is a relatively unspecific modification that correlates with transcriptional activity. It is deposited by histone acetyltransferases (HATs), such as p300 or CBP, which play direct roles in gene activation [69]. Furthermore, acetylation affects chromatin accessibility as it neutralizes the positive charge of histone tail residues, reducing their interaction with nucleosomal DNA. Conversely, histone methylation is a more specific modification deposited by histone methyltransferases and, sometimes, has distinct regulatory roles. For example, H3K9me3 is associated with repressed chromatin [64], while the same modification on lysine 4 (H3K4me3) is associated with active promoters [70]. Histone modifications are recognized by different reader proteins, which in turn recruit cofactors that have activating or repressive functions.

The exit from naive pluripotency provides insights into how histone modifications might be involved in the co-ordination of cell fate transitions. Active enhancers can be identified by the combination of multiple specific epigenetic marks: H3K27ac (acetylation of the 27th lysine residue of histone H3 [71]) and H3K4me1 (monomethylation of the 4th lysine residue of histone H3). Recently, H2BNTac (multi-site N-terminal acetylation of histone H2B) has also seen use as a marker of enhancers [72]. While H3K27ac is the main marker of active enhancers, preventing H3K27ac deposition at enhancers has a negligible effect on transcription in mESCs [59,60]. *De novo* H3K27 acetylation is also not an absolute prerequisite up-regulation of genes during naive exit [59]. This suggests an inherent robustness to, and independence of, the regulatory networks that work alongside the *de facto* deposited mark.

Similar to chromatin accessibility, the presence of H3K27ac does not always reflect intrinsic enhancer activity. The enhancer cluster regulating *Fgf5* during the exit from naive pluripotency illustrates this complexity. There, multiple enhancers contribute to gene activation at different time points [73]. Each of the enhancers shows similar levels of H3K27ac; however, none of the elements has strong intrinsic enhancer activity, and only in co-operation are these elements able to activate the expression of *Fgf5* during the exit from naive pluripotency (discussed in further detail below).

H3K4me1 is primarily catalyzed by the methyltransferases KMT2C and KMT2D (previously named MLL3 and MLL4) [74]. Despite mutations in both KMT2C and KMT2D being embryonic lethal beyond the post-implantation stages [75], these mutations do not significantly reduce global levels of H3K4me1. In mESCs, the deposition of H3K4me1 by KMT2C/D is not necessary for maintaining naive pluripotency but might be crucial for sustaining differentiation, with one study suggesting that KMT2D is needed for H3K27ac deposition by p300/CBP through H3K4me1-mediated priming and that this is required for mESC differentiation *in vitro* [76]. Refuting this proposed link, most, if not all, enhancers can gain H3K27ac independent of KMT2C/D-mediated H3K4me1, and their knockout has no significant effect on gene expression [57]. KMT2C/D have also been shown to have functions independent of their catalytic activity, as cells expressing catalytically deficient KMT2C/D, as opposed to their complete knockout, show only slight effects on transcription [77]. KMT2D is needed for the exit of naive pluripotency, but this is independent of its catalytic activity [78].

While H3K4me1 on its own might be dispensable for enhancer activation, it might play a supportive role in cell state transitions, ensuring that the correct genes are activated [58]. H3K4me1 aids in enhancer–promoter interactions and enhancer-driven transcription during differentiation, primarily mediated by KMT2C/D and KMT2B [79], which could be crucial for long-range chromatin interactions [80].

Differentiation relies on the repression of genes outside of the intended lineage. This is ensured in part by Polycomb-repressive complex 2 (PRC2)-mediated deposition of H3K27me3. In naive pluripotency, H3K27me3 is widespread across the genome [81]. In the absence of the mark, naive pluripotency can be maintained, but differentiation is impaired [61–63]. This stands in contrast with the proposedly negligible effect of H3K27ac loss [59,60], suggesting a dominant role for H3K27me3 in regulating enhancers, corroborated by its absence on repressed enhancers leading to aberrant gene expression [55]. PRC2 catalytic subunit EZH2 deletion is embryonically lethal post-implantation [82], as are deletions of subunits EED [83] and SUZ12 [84], emphasizing that, while H3K27me3 is not a requirement for productive gene regulatory networks per se, it contributes to proper spatiotemporal control of genes.

PRC2 works together with Polycomb-repressive complex 1 (PRC1) to silence genes and to organize chromatin. PRC1 mediates H2AK119 monoubiquitylation, which contributes to transcriptional repression by restricting RNA polymerase II initiation [65]. Two PRC1 configurations exist, and while naive pluripotency can be maintained without either, their simultaneous loss leads to differentiation [66]. The two PRC complexes are also important for chromatin architecture [85], facilitating enhancer:promoter contacts prior to transcription [86], likely independent of their enzymatic activity [87–89].

Cell state transitions involve priming the genome for expression of new genes and simultaneously preventing their misexpression. Enhancers bivalently marked by H3K4me1 and H3K27me3 can be found during naive pluripotency and are linked to developmentally relevant genes [90]. Such enhancers are proposed to be ‘poised’, inactive but prepared for activation or repression in cell state transitions downstream of the state in which they were first established. Poised enhancers are associated with orphan CpG islands, which suggests that bivalency could have a structural function by acting as tethering elements between poised enhancers and their cognate promoters [91]. Put differently, when not part of deliberate repression, bivalency could serve to establish the topology of the chromatin landscape needed for somatic cell differentiation in a PRC-dependent manner, which is especially important in terms of species-specific developmental control [86,91]. Further studies of naive exit will help elucidate how bivalent enhancers help cells navigate between activation and repression during cell state transitions.

### DNA methylation

DNA methylation as a layer of gene regulation has a debated function in enhancer activity, but the exit from naive pluripotency could help to elucidate some of its functions. DNA methylation is depleted from the zygotic genome as pluripotency is attained and is reassembled to poise cells for differentiation as naive pluripotency is established. The naive state is marked by low global levels of DNA methylation, which increases upon differentiation [92,93]. Early studies suggested that loss of DNA methylation impairs differentiation potential of mESCs cultured under serum conditions, where signaling pathways are highly heterogeneous [94]. However, more recent evidence obtained from defined 2i/LIF conditions suggests that DNA methylation is largely dispensable for the exit from naive pluripotency and does not significantly impair differentiation potential [95].

DNA methylation in mammals occurs primarily as 5′-methylcytosine within CpG dinucleotides and is associated with compaction of DNA, thereby potentially preventing the binding of transcriptional activators. Loss of DNA methylation has little effect on mESC self-renewal and gene expression patterns, and can only be implied to be relevant in the formation of primordial germ cells [96]. The lack of methylation induces epigenetic changes primarily to enhancers and simultaneously extends the temporal window for germ cell differentiation [95]. This might reflect an overarching lack in the ability to establish strong restrictive epigenetic states, as a genome without methylation could likely see more promiscuous activation. Comparatively, the loss of TET enzyme function leads to widespread hypermethylation, particularly at gene promoters, and impairs the differentiation capacity of mESCs, as seen in embryoid bodies and teratomas [97]. Methylation can directly prevent DNA binding of proteins that recognize specific DNA motifs or create binding sites for factors that preferentially recognize methylated DNA [98]. Only a very small subset of enhancers is regulated through DNA methylation [99], hinting that DNA methylation is not a common tool used by the cell to control gene expression, but that it might be important in specific regulatory and developmental contexts [67].

### Enhancer-promoter proximity

Often located tens to hundreds of kilobases away from their target promoters, enhancers circumvent separation in two-dimensional space by acting in three dimensions. One way to overcome separation by large genomic distances is to displace the genome, keeping two gene regulatory elements apart. This can be achieved through cohesin-mediated loop extrusion [100]. Cohesin proteins form a ring-shaped complex that acts as a molecular motor. When loaded onto chromatin, the DNA is extruded through the ring structure and thereby distant elements are brought into proximity to each other. The process is dynamic, and the constant shuttling of DNA could facilitate enhancer–promoter interactions [101]. It is also conceivable that, rather than through creating order, cohesin instead primarily functions as a disruptor of chromatin interactions, preventing excessive compaction to ensure that regulatory elements remain free to interact [102].

The Mediator complex is another key regulator of enhancer–promoter interactions, acting as a molecular bridge between TFs at enhancers and RNA polymerase II at promoters. Rapid depletion of Mediator leads to reduced enhancer-promoter contacts, decreased gene expression, and lowered binding of cohesin at enhancers [103]. Possibly, Mediator acts to strengthen and sustain cohesin-induced loops to ensure robust gene regulation.

While many long-distance enhancers might depend on cohesin [104], acute loss of cohesin has only minor effects on enhancer-promoter interactions and gene expression in steady state [105–107]. It was argued that, during differentiation, cohesin-mediated loop extrusion might be important to establish enhancer-promoter communication, but once established, loop extrusion might no longer be needed. However, depletion of the cohesin processivity factor Nipbl during differentiation has little effect on gene expression despite loss of most cohesin-mediated loops [108].

Several cases challenge the model of cohesin-mediated looping being the primary mechanism for enhancer–promoter interactions. For instance, the zone of polarizing activity regulatory sequence (ZRS) enhancer, which regulates Shh expression during limb development, functions over a megabase away from its target promoter [109,110]. Although promoter-enhancer looping has been proposed as the main activating step [111], studies suggest that Shh expression can persist even when the physical distance between the enhancer and promoter increases, indicating that other structural or regulatory mechanisms are at play [112]. Similarly, *Sox2* transcription can occur without detectable enhancer-promoter interactions, questioning the necessity of stable loops for gene activation [113]. Rather than acting as a strict requirement, looping may play an early role in establishing genome topology, but once regulatory hubs form, transcription might continue independently of physical enhancer–promoter proximity [114,115].

High-resolution studies of the three-dimensional genome architecture have revealed so-called microcompartments. Within these, enhancers and promoters might connect independently of cohesin, since their interactions are largely unaffected by both transcriptional inhibition and loss of loop extrusion [116]. At the microcompartmental scale, enhancers show widespread interactions with both, other enhancers and target promoters, suggesting gene regulation is, at least in part, a product of local networks of collaborating regulatory elements. Accordingly, the co-operative action of multiple enhancers has been suggested to play a direct role in supporting cohesin-independent gene activation [108,117].

### Enhancer cooperativity

Throughout differentiation, the enhancer network is continually rewired to promote novel gene regulatory networks that sustain specific cell states. This process is far more complex than a simple ‘enhancer on = gene on’ or ‘enhancer off = gene off’ model. Instead, as cells transition between states, enhancers are selectively activated, repressed, or repurposed, resulting in the up-regulation of some genes while others are silenced.

Crucially, enhancers do not act in isolation; they function within cooperative regulatory landscapes, where each element affects the function of the others. This coordinated enhancer remodeling ensures precise transcriptional control, allowing cells to establish and maintain distinct identities.

Enhancers that collaborate can carry out different roles. In the regulation of *Klf4* during pluripotency, several elements interact in a hierarchical manner to sustain its expression. While disrupting a single enhancer only has a mild effect, the removal of multiple enhancers leads to a significant reduction in *Klf4* expression, highlighting functional redundancy within the regulatory network [118]. Even super-enhancers are often modular and consist of multiple individual enhancer elements spread out over multiple kilobases. Five enhancer elements collaboratively activate *Fgf5* expression during the exit from naive pluripotency, with four intergenic elements forming a super-enhancer that induces *Fgf5* at distinct times. The fifth intronic enhancer consistently amplifies the expression of *Fgf5*, resulting in a strong, super-additive induction [73]. Enhancer cooperativity can significantly mitigate the reduction in gene activation caused by increased genomic distances, particularly when a weaker enhancer is positioned between a strong enhancer and its target promoter [117]. Therefore, weak enhancers can take on a facilitator role to help strong enhancers communicate with their target promoter efficiently [46]. The extent to which facilitator elements play a role in all enhancer-promoter communications has yet to be established.

Not all regulatory elements associated with a gene act to promote its expression: Enhancers also function together with silencers, and a developmentally relevant example of this is *Cdx2*, where a single TF motif turns from silencing to enhancing [119]. Enhancer interplay might establish the above-mentioned microcompartments and thereby increase the interaction of distal enhancers with the target promoter independent of cohesin [108]. This regulatory coordination is especially critical during cell state transitions, where chromatin structure is rapidly reorganized to establish new transcriptional programs. Recent findings reveal that as cells exit naive pluripotency, enhancerpromoter interactions undergo large-scale reconfiguration through the formation of multiway chromatin hubs, which bring together distant enhancers and promoters into shared regulatory environments [36]. These hubs provide a structural framework for genes associated with pluripotency exit to establish long-range interactions with emerging enhancers, suggesting that spatial genome architecture plays a key role in facilitating transcriptional transitions. Taken together, enhancers don’t work in isolation, and their impact can only be examined as part of a larger regulatory framework.

The exit from naive pluripotency presents an ideal model to study enhancer mechanisms, as it involves widespread chromatin reorganization, enhancer activation, and long-range genomic interactions necessary for lineage specification. By examining how enhancers integrate into multiway chromatin hubs during this transition, we can gain critical insights into how regulatory elements shape gene expression dynamics in development.

## Discussion

The exit from naive pluripotency offers an invaluable opportunity for the holistic understanding of enhancer mechanistics. mESCs are easily maintained *in vitro*, highly amenable to perturbations, and their intrinsic differentiation trajectory recapitulates the rewiring of the enhancer landscape that allows for cell fate transitions during development. Most other developmental cell fate transitions are multi-pronged, and while successes have been made in probing regulatory elements in multicellular settings [120], the multi-omic approaches needed to reliably probe the complexity of enhancer functionality in heterogeneous systems are currently underdeveloped. mESCs circumvent the drawbacks of more complex systems, as they provide a near limitless amount of cells undergoing a single, developmentally relevant, cell state transition. Therefore, the exit of naive pluripotency remains the prime choice for studies that tease apart how enhancers work in determining cell fates in mammals.

Many aspects of gene regulation in development are still poorly understood, and it is not quite clear how individual factors studied in one cellular context contribute to cell fate transitions across multiple states. Despite its role in redirecting OCT4 binding to differentiation-specific enhancers, OTX2 is not vital for the exit of naive pluripotency. mESCs null for OTX2 display deficiencies in differentiating but will readily integrate into blastocysts and give rise to chimeric embryos [121]. Highly chimeric embryos show a phenotype consistent with the effect of its *in vivo* deletion, where the loss of OTX2 leads to the failure in the development of head structures [122]. This highlights that the effects of the lack of OTX2 are not caused by the lack of a single-cell fate decision, but through the compound effects that the absence of OTX2 has on anterior neural differentiation during development. Thus, pinpointing a distinct causal effect in development is difficult, as between one stage and another, a cell state can be largely indistinguishable from the wild type and a deleterious phenotype would only become apparent through the compound effects of subsequent transitions.

The robustness of cell state transitions and the transcription factor networks that drive them are a central theme in studies utilizing the exit of naive pluripotency as a model [123]. No single factor solely responsible for this cell state transition has been identified so far, despite genome-wide screens having been performed to saturation [15]. Transitioning between the naive and formative pluripotent state involves the modulation of multiple signaling pathways, including FGF/ERK, WNT/β-catenin, LIF, Notch, and mTORC1 [15]. Accordingly, the lack of activators and repressors of these pathways reproducibly correlates to defects in pluripotency and differentiation.

TCF7L1 recurrently appears as a target in loss of function screens of naive exit [124]. TCF7L1 is a TF that acts as a repressor of WNT target genes and its ablation replaces WNT activation in culture of mESCs [125]. Since WNT signaling is needed to uphold pluripotency and stemness in culture, which is mediated through repression of TCF7L1 [126], it is no surprise that TCF7L1 promotes naive exit. In the absence of TCF7L1, naive exit is delayed but not halted. This is true also for other central signaling mediators [15]. Only through the concurrent knockout of components belonging to different signaling pathways can ESCs be arrested in pluripotency. This is demonstrated in the triple knockout of TFs *Etv5*, *Rbpj*, and *Tcf7l1* (facilitating FGF/ERK, Notch, and WNT signaling, respectively) arresting mESCs in naive pluripotency [127]. This highlights that the concerted efforts of different pathways are necessary to drive naive exit and that the enhancer landscape can be rewired even in the case of perturbations.

As our understanding of enhancer function deepens, future research must address how enhancer networks integrate multiple levels of regulatory input to generate stable, but flexible, transcriptional states. Advances in synthetic biology and genome engineering now offer the potential to build regulatory environments from the bottom up. By leveraging mESC models, enhancers can be introduced into synthetic loci and functionally probed in a developmental context, allowing researchers to systematically define enhancer logic and regulatory architectures in ways previously impossible. Understanding how enhancer landscapes can be manipulated *in vivo* will not only advance developmental biology but may also pave the way for precise engineering of cell fate transitions in regenerative medicine and biotechnological innovations.

**Perspectives**

- Importance for the field: Enhancers are the main gene regulatory elements that convey spatiotemporal control of gene expression, specifically in development. The exit of naive pluripotency provides an accessible and versatile platform to study enhancer mechanistics during cell state transitions.
- Current thinking in the field: The embryo and systems modeling embryonic development are not ideal to understand how transcriptional changes are regulated. In larger gene regulatory networks and in enhancer clusters, deficiencies can often be overcome by other redundant factors.
- Future directions: Novel tools including developments in synthetic biology, targeted protein degradation, and dual screening strategies in combination with the exit from naive pluripotency have the potential to deepen our understanding of transcriptional regulation during cell state transitions.

## Abbreviations

H2BNTac: multi-site N-terminal acetylation of histone H2B
H3K27ac: acetylation of lysine 27 on histone H3
H3K4me1: methylation of lysine 4 on histone H3
H3K9me3: trimethylation of lysine 9 of histone 3
H3K27me3: trimethylation of lysine 27 on histone H3
LIF: leukemia inhibitory factor
PRC1: Polycomb-repressive complex 1
PRC2: Polycomb-repressive complex 2
TF: transcription factor
2i: two small-molecule inhibitors
mESC: mouse embryonic stem cell

## References

[1] Gaiti F. Jindrich K. Fernandez-Valverde S.L. Roper K.E. Degnan B.M. Tanurdžić M . Landscape of histone modifications in a sponge reveals the origin of animal cis-regulatory complexity Elife 6 10.7554/eLife.22194 PMC5429095 28395144

[2] Dejosez M. Dall’Agnese A. Ramamoorthy M. Platt J. Yin X. Hogan M et al 2023 Regulatory architecture of housekeeping genes is driven by promoter assemblies Cell Rep 42 112505 10.1016/j.celrep.2023.112505 37182209 PMC10329844

[3] Liu T.T. Ou F. Belk J.A. Bagadia P. Anderson D.A. Durai V et al 2023 Cis interactions in the Irf8 locus regulate stage-dependent enhancer activation Genes Dev. 37 291 302 10.1101/gad.350339.122 36990511 PMC10153461

[4] Gschwind A.R. Mualim K.S. Karbalayghareh A. Sheth M.U. Dey K.K. Jagoda E. et al 2023 An encyclopedia of enhancer-gene regulatory interactions in the human genome bioRxiv 2023, 2023.11.09.563812 10.1101/2023.11.09.563812 38014075 PMC10680627

[5] Osterwalder M. Barozzi I. Tissières V. Fukuda-Yuzawa Y. Mannion B.J. Afzal S.Y. et al 2018 Enhancer redundancy provides phenotypic robustness in mammalian development Nature 554 239 243 10.1038/nature25461 29420474 PMC5808607

[6] Frankel N. Davis G.K. Vargas D. Wang S. Payre F. Stern DL 2010 Phenotypic robustness conferred by apparently redundant transcriptional enhancers Nature 466 490 493 10.1038/nature09158 20512118 PMC2909378

[7] Hong J.W. Hendrix D.A. Levine M.S 2008 Shadow enhancers as a source of evolutionary novelty Science 321 1314 1314 10.1126/science.1160631 18772429 PMC4257485

[8] Perry M.W. Boettiger A.N. Bothma J.P. Levine M 2010 Shadow enhancers foster robustness of drosophila gastrulation Curr. Biol. 20 1562 1567 10.1016/j.cub.2010.07.043 20797865 PMC4257487

[9] Ahituv N. Zhu Y. Visel A. Holt A. Afzal V. Pennacchio L.A. et al 2007 Deletion of ultraconserved elements yields viable mice PLoS Biol. 5 e234 10.1371/journal.pbio.0050234 17803355 PMC1964772

[10] Dickel D.E. Ypsilanti A.R. Pla R. Zhu Y. Barozzi I. Mannion B.J et al 2018 Ultraconserved enhancers are required for normal development Cell 172 491 499 10.1016/j.cell.2017.12.017 29358049 PMC5786478

[11] Nolte M.J. Wang Y. Deng J.M. Swinton P.G. Wei C. Guindani M. et al 2014 Functional analysis of limb transcriptional enhancers in the mouse Evol. Dev. 16 207 223 10.1111/ede.12084 24920384 PMC4130292

[12] Kvon E.Z. Waymack R. Gad M. Wunderlich Z 2021 Enhancer redundancy in development and disease Nat. Rev. Genet. 22 324 336 10.1038/s41576-020-00311-x 33442000 PMC8068586

[13] Boroviak T. Loos R. Bertone P. Smith A. Nichols J 2014 The ability of inner-cell-mass cells to self-renew as embryonic stem cells is acquired following epiblast specification Nat. Cell Biol. 16 516 528 10.1038/ncb2965 24859004 PMC4878656

[14] Kalkan T. Smith A 2014 Mapping the route from naive pluripotency to lineage specification Phil. Trans. R. Soc. B 369 20130540 10.1098/rstb.2013.0540 25349449 PMC4216463

[15] Lackner A. Sehlke R. Garmhausen M. Giuseppe Stirparo G. Huth M. Titz-Teixeira F. et al 2021 Cooperative genetic networks drive embryonic stem cell transition from naïve to formative pluripotency EMBO J. 40 e105776 10.15252/embj.2020105776 33687089 PMC8047444

[16] Jayaram S. Romeike M. Buecker C 2023 The asynchrony in the exit from naive pluripotency cannot be explained by differences in the cell cycle phase 2023 Developmental Biology 10.1101/2023.09.15.557731

[17] Mulas C. Stammers M. Salomaa S.I. Heinzen C. Suter D.M. Smith A. et al 2024 ERK signalling eliminates Nanog and maintains Oct4 to drive the formative pluripotency transition Development (Rome) 151 dev203106 10.1242/dev.203106 39069943

[18] Waisman A. Sevlever F. Elías Costa M. Cosentino M.S. Miriuka S.G. Ventura A.C. et al 2019 Cell cycle dynamics of mouse embryonic stem cells in the ground state and during transition to formative pluripotency Sci. Rep. 9 8051 10.1038/s41598-019-44537-0 31142785 PMC6541595

[19] Saykali B. Tran A.D. Cornwell J.A. Caldwell M.A. Sangsari P.R. Morgan N.Y. et al 2025 Lineage-specific CDK activity dynamics characterize early mammalian development Cell Rep. 44 115558, S2211-1247(25)00329-8 10.1016/j.celrep.2025.115558 40220290 PMC12070373

[20] Passaro F. De Martino I. Zambelli F. Di Benedetto G. Barbato M. D’Erchia A.M. et al 2021 YAP contributes to DNA methylation remodeling upon mouse embryonic stem cell differentiation J. Biol. Chem. 296 100138 10.1074/jbc.RA120.015896 33268382 PMC7948423

[21] Shahbazi M.N. Scialdone A. Skorupska N. Weberling A. Recher G. Zhu M. et al 2017 Pluripotent state transitions coordinate morphogenesis in mouse and human embryos Nature 552 239 243 10.1038/nature24675 29186120 PMC5768241

[22] Ulfig A. Jakob U 2024 Redox heterogeneity in mouse embryonic stem cells individualizes cell fate decisions Dev. Cell 59 2118 2133 10.1016/j.devcel.2024.07.008 39106861 PMC11338707

[23] Ochiai H. Sugawara T. Sakuma T. Yamamoto T 2014 Stochastic promoter activation affects Nanog expression variability in mouse embryonic stem cells Sci. Rep. 4 7125 10.1038/srep07125 25410303 PMC4238020

[24] Luo Y. He J. Xu X. Sun M.-A. Wu X. Lu X. et al 2018 Integrative single-cell omics analyses reveal epigenetic heterogeneity in mouse embryonic stem cells PLOS Comput. Biol. 14 e1006034 10.1371/journal.pcbi.1006034 29561833 PMC5862410

[25] Carbognin E. Carlini V. Panariello F. Chieregato M. Guerzoni E. Benvegnù D. et al 2023 Esrrb guides naive pluripotent cells through the formative transcriptional programme Nat. Cell Biol. 25 643 657 10.1038/s41556-023-01131-x 37106060 PMC7614557

[26] Semrau S. Goldmann J.E. Soumillon M. Mikkelsen T.S. Jaenisch R. van Oudenaarden A. 2017 Dynamics of lineage commitment revealed by single-cell transcriptomics of differentiating embryonic stem cells Nat. Commun. 8 1096 10.1038/s41467-017-01076-4 29061959 PMC5653659

[27] Dunn S.J. Martello G. Yordanov B. Emmott S. Smith AG 2014 Defining an essential transcription factor program for naïve pluripotency Science 344 1156 1160 10.1126/science.1248882 24904165 PMC4257066

[28] Buecker C. Srinivasan R. Wu Z. Calo E. Acampora D. Faial T. et al 2014 Reorganization of enhancer patterns in transition from naive to primed pluripotency Cell Stem Cell 14 838 853 10.1016/j.stem.2014.04.003 24905168 PMC4491504

[29] Yang S.-H. Kalkan T. Morissroe C. Marks H. Stunnenberg H. Smith A et al 2014 Otx2 and Oct4 drive early enhancer activation during embryonic stem cell transition from naive pluripotency Cell Rep 7 1968 1981 10.1016/j.celrep.2014.05.037 24931607 PMC4074343

[30] Qiu D. Ye S. Ruiz B. Zhou X. Liu D. Zhang Q et al 2015 Klf2 and Tfcp2l1, Two Wnt/β-Catenin targets, act synergistically to induce and maintain naive pluripotency Stem Cell Reports 5 314 322 10.1016/j.stemcr.2015.07.014 26321140 PMC4618593

[31] Wang X. Wang X. Zhang S. Sun H. Li S. Ding H. et al 2019 The transcription factor TFCP2L1 induces expression of distinct target genes and promotes self-renewal of mouse and human embryonic stem cells Journal of Biological Chemistry 294 6007 6016 10.1074/jbc.RA118.006341 30782842 PMC6463713

[32] Respuela P. Nikolić M. Tan M. Frommolt P. Zhao Y. Wysocka J et al 2016 Foxd3 Promotes exit from naive pluripotency through enhancer decommissioning and inhibits germline specification Cell Stem Cell 18 118 133 10.1016/j.stem.2015.09.010 26748758 PMC5048917

[33] Waisman A. Sevlever F. Saulnier D. Francia M. Blanco R. Amín G. et al 2024 The transcription factor OCT6 promotes the dissolution of the naïve pluripotent state by repressing Nanog and activating a formative state gene regulatory network Sci. Rep. 14 10420 10.1038/s41598-024-59247-5 38710730 PMC11074312

[34] Yang S.H. Andrabi M. Biss R. Murtuza Baker S. Iqbal M. Sharrocks A.D 2019 ZIC3 Controls the transition from naive to primed pluripotency Cell Rep 27 3215 3227 10.1016/j.celrep.2019.05.026 31189106 PMC6581693

[35] Chen A.F. Liu A.J. Krishnakumar R. Freimer J.W. DeVeale B. Blelloch R 2018 GRHL2-Dependent enhancer switching maintains a pluripotent stem cell transcriptional subnetwork after exit from naive pluripotency Cell Stem Cell 23 226 238 10.1016/j.stem.2018.06.005 30017589 PMC6456389

[36] Lando D. Ma X. Cao Y. Jartseva A. Stevens T.J. Boucher W et al 2024 Enhancer-promoter interactions are reconfigured through the formation of long-range multiway hubs as mouse ES cells exit pluripotency Mol. Cell 84 1406 1421 10.1016/j.molcel.2024.02.015 38490199 PMC7616059

[37] Betschinger J. Nichols J. Dietmann S. Corrin P.D. Paddison P.J. Smith A 2013 Exit from pluripotency is gated by intracellular redistribution of the bHLH transcription factor Tfe3 Cell 153 335 347 10.1016/j.cell.2013.03.012 23582324 PMC3661979

[38] Wei Z. Gao F. Kim S. Yang H. Lyu J. An W et al 2013 Klf4 organizes long-range chromosomal interactions with the oct4 locus in reprogramming and pluripotency Cell Stem Cell 13 36 47 10.1016/j.stem.2013.05.010 23747203

[39] Dhaliwal N.K. Miri K. Davidson S. Tamim El Jarkass H. Mitchell J.A 2018 KLF4 Nuclear Export Requires ERK Activation and Initiates Exit from Naive Pluripotency Stem Cell Reports 10 1308 1323 10.1016/j.stemcr.2018.02.007 29526737 PMC6000723

[40] Wei F. Schöler H.R. Atchison M.L 2007 Sumoylation of Oct4 enhances its stability, DNA binding, and transactivation J. Biol. Chem. 282 21551 21560 10.1074/jbc.M611041200 17525163

[41] Abulaiti X. Zhang H. Wang A. Li N. Li Y. Wang C et al 2017 Phosphorylation of Threonine^343^ Is Crucial for OCT4 Interaction with SOX2 in the maintenance of mouse embryonic stem cell pluripotency Stem Cell Reports 9 1630 1641 10.1016/j.stemcr.2017.09.001 28988986 PMC5829306

[42] Jang H. Kim T.W. Yoon S. Choi S.-Y. Kang T.-W. Kim S.-Y. et al 2012 O-GlcNAc regulates pluripotency and reprogramming by directly acting on core components of the pluripotency network Cell Stem Cell 11 62 74 10.1016/j.stem.2012.03.001 22608532

[43] Hihara S. Pack C.-G. Kaizu K. Tani T. Hanafusa T. Nozaki T et al 2012 Local nucleosome dynamics facilitate chromatin accessibility in living mammalian cells Cell Rep 2 1645 1656 10.1016/j.celrep.2012.11.008 23246002

[44] Sahu B. Hartonen T. Pihlajamaa P. Wei B. Dave K. Zhu F. et al 2022 Sequence determinants of human gene regulatory elements Nat. Genet. 54 283 294 10.1038/s41588-021-01009-4 35190730 PMC8920891

[45] Peng T. Zhai Y. Atlasi Y. Ter Huurne M. Marks H. Stunnenberg H.G. et al 2020 STARR-seq identifies active, chromatin-masked, and dormant enhancers in pluripotent mouse embryonic stem cells Genome Biol. 21 243 10.1186/s13059-020-02156-3 32912294 PMC7488044

[46] Blayney J.W. Francis H. Rampasekova A. Camellato B. Mitchell L. Stolper R et al 2023 Super-enhancers include classical enhancers and facilitators to fully activate gene expression Cell 186 5826 5839 10.1016/j.cell.2023.11.030 38101409 PMC10858684

[47] Dupont C. Chahar D. Trullo A. Gostan T. Surcis C. Grimaud C. et al 2023 Evidence for low nanocompaction of heterochromatin in living embryonic stem cells EMBO J. 42 e110286 10.15252/embj.2021110286 37082862 PMC10267699

[48] Joron K. Viegas J.O. Haas-Neill L. Bier S. Drori P. Dvir S. et al 2023 Fluorescent protein lifetimes report densities and phases of nuclear condensates during embryonic stem-cell differentiation Nat. Commun. 14 4885 10.1038/s41467-023-40647-6 37573411 PMC10423231

[49] Cho W.-K. Spille J.-H. Hecht M. Lee C. Li C. Grube V. et al 2018 Mediator and RNA polymerase II clusters associate in transcription-dependent condensates Science 361 412 415 10.1126/science.aar4199 29930094 PMC6543815

[50] Klingberg T. Wachter I. Pancholi A. Gohar Y. Kumar P. Sobucki M et al 2023 Transcriptional clusters follow a conserved condensation-dispersal sequence during stem cell differentiation Dev. Biol. (NY) 2023.07.04.547621 10.1101/2023.07.04.547621

[51] Murtha M. Strino F. Tokcaer-Keskin Z. Sumru Bayin N. Shalabi D. Xi X. et al 2015 Comparative FAIRE-seq analysis reveals distinguishing features of the chromatin structure of ground state- and primed-pluripotent cells Stem Cells 33 378 391 10.1002/stem.1871 25335464 PMC4304912

[52] Ahmed K. Dehghani H. Rugg-Gunn P. Fussner E. Rossant J. Bazett-Jones DP 2010 Global chromatin architecture reflects pluripotency and lineage commitment in the early mouse embryo PLOS ONE 5 e10531 10.1371/journal.pone.0010531 20479880 PMC2866533

[53] Bultman S. Gebuhr T. Yee D. La Mantia C. Nicholson J. Gilliam A. et al 2000 A Brg1 null mutation in the mouse reveals functional differences among mammalian SWI/SNF complexes Mol. Cell 6 1287 1295 10.1016/s1097-2765(00)00127-1 11163203

[54] King H.W. Klose R.J 2017 The pioneer factor OCT4 requires the chromatin remodeller BRG1 to support gene regulatory element function in mouse embryonic stem cells Elife 6 e22631 10.7554/eLife.22631 28287392 PMC5400504

[55] Trovato M. Bunina D. Yildiz U. Fernandez-Novel Marx N. Uckelmann M. Levina V. et al 2024 Histone H3.3 lysine 9 and 27 control repressive chromatin at cryptic enhancers and bivalent promoters Nat. Commun. 15 7557 10.1038/s41467-024-51785-w 39214979 PMC11364623

[56] Zhang J. Donahue G. Gilbert M.B. Lapidot T. Nicetto D. Zaret KS 2024 Distinct H3K9me3 heterochromatin maintenance dynamics govern different gene programmes and repeats in pluripotent cells Nat. Cell Biol. 26 2115 2128 10.1038/s41556-024-01547-z 39482359 PMC12908651

[57] Boileau R.M. Chen K.X. Blelloch R 2023 Loss of MLL3/4 decouples enhancer H3K4 monomethylation, H3K27 acetylation, and gene activation during embryonic stem cell differentiation Genome Biol. 24 41 10.1186/s13059-023-02883-3 36869380 PMC9983171

[58] Bleckwehl T. Crispatzu G. Schaaf K. Respuela P. Bartusel M. Benson L. et al 2021 Enhancer-associated H3K4 methylation safeguards in vitro germline competence Nat. Commun. 12 5771 10.1038/s41467-021-26065-6 34599190 PMC8486853

[59] Sankar A. Mohammad F. Sundaramurthy A.K. Wang H. Lerdrup M. Tatar T. et al 2022 Histone editing elucidates the functional roles of H3K27 methylation and acetylation in mammals Nat. Genet. 54 754 760 10.1038/s41588-022-01091-2 35668298

[60] Zhang T. Zhang Z. Dong Q. Xiong J. Zhu B 2020 Histone H3K27 acetylation is dispensable for enhancer activity in mouse embryonic stem cells Genome Biol. 21 45 10.1186/s13059-020-01957-w 32085783 PMC7035716

[61] Miller S.A. Damle M. Kim J. Kingston RE 2021 Full methylation of H3K27 by PRC2 is dispensable for initial embryoid body formation but required to maintain differentiated cell identity Development 148 dev196329 10.1242/dev.196329 33688077 PMC8077505

[62] Shan Y. Liang Z. Xing Q. Zhang T. Wang B. Tian S. et al 2017 PRC2 specifies ectoderm lineages and maintains pluripotency in primed but not naïve ESCs Nat. Commun. 8 672 10.1038/s41467-017-00668-4 28939884 PMC5610324

[63] van Mierlo G Dirks R.A.M. De Clerck L. Brinkman A.B. Huth M. Kloet S.L et al 2019 Integrative Proteomic Profiling Reveals PRC2-dependent epigenetic crosstalk maintains ground-state pluripotency Cell Stem Cell 24 123 137 10.1016/j.stem.2018.10.017 30472157

[64] Nicetto D. Zaret KS 2019 Role of H3K9me3 heterochromatin in cell identity establishment and maintenance Curr. Opin. Genet. Dev. 55 1 10 10.1016/j.gde.2019.04.013 31103921 PMC6759373

[65] Dobrinić P. Szczurek A.T. Klose RJ 2021 PRC1 drives Polycomb-mediated gene repression by controlling transcription initiation and burst frequency Nat. Struct. Mol. Biol. 28 811 824 10.1038/s41594-021-00661-y 34608337 PMC7612713

[66] Zepeda-Martinez J.A. Pribitzer C. Wang J. Bsteh D. Golumbeanu S. Zhao Q. et al 2020 Parallel PRC2/cPRC1 and vPRC1 pathways silence lineage-specific genes and maintain self-renewal in mouse embryonic stem cells Sci. Adv. 6 eaax5692 10.1126/sciadv.aax5692 32270030 PMC7112768

[67] Kreibich E. Krebs AR 2023 Relevance of DNA methylation at enhancers for the acquisition of cell identities FEBS Lett. 597 1805 1817 10.1002/1873-3468.14686 37343149

[68] Murphy A.E. Askarova A. Lenhard B. Skene N.G. Marzi S.J 2025 Predicting gene expression from histone marks using chromatin deep learning models depends on histone mark function, regulatory distance and cellular states Nucleic Acids Res. 53 gkae1212 10.1093/nar/gkae1212 39660643 PMC11879020

[69] Verdone L. Caserta M. Di Mauro E 2005 Role of histone acetylation in the control of gene expression Biochem. Cell Biol. 83 344 353 10.1139/o05-041 15959560

[70] Yu H. Lesch BJ 2024 Functional Roles of H3K4 methylation in transcriptional regulation Mol. Cell. Biol. 44 505 515 10.1080/10985549.2024.2388254 39155435 PMC11529435

[71] Creyghton M.P. Cheng A.W. Welstead G.G. Kooistra T. Carey B.W. Steine E.J. et al 2010 Histone H3K27ac separates active from poised enhancers and predicts developmental state Proc. Natl. Acad. Sci. U.S.A 107 21931 21936 10.1073/pnas.1016071107 21106759 PMC3003124

[72] Narita T. Higashijima Y. Kilic S. Liebner T. Walter J. Choudhary C 2023 Acetylation of histone H2B marks active enhancers and predicts CBP/p300 target genes Nat. Genet. 55 679 692 10.1038/s41588-023-01348-4 37024579 PMC10101849

[73] Thomas H.F. Kotova E. Jayaram S. Pilz A. Romeike M. Lackner A et al 2021 Temporal dissection of an enhancer cluster reveals distinct temporal and functional contributions of individual elements Mol. Cell 81 969 982 10.1016/j.molcel.2020.12.047 33482114

[74] Van H.T. Xie G. Dong P. Liu Z. Ge K 2024 KMT2 Family of H3K4 Methyltransferases: Enzymatic Activity-dependent and -independent Functions J. Mol. Biol 436 168453 10.1016/j.jmb.2024.168453 38266981 PMC10957308

[75] Ashokkumar D. Zhang Q. Much C. Bledau A.S. Naumann R. Alexopoulou D. et al 2020 MLL4 is required after implantation, whereas MLL3 becomes essential during late gestation Development 147 dev186999 10.1242/dev.186999 32439762

[76] Wang C. Lee J.-E. Lai B. Macfarlan T.S. Xu S. Zhuang L. et al 2016 Enhancer priming by H3K4 methyltransferase MLL4 controls cell fate transition Proc. Natl. Acad. Sci. U.S.A 113 11871 11876 10.1073/pnas.1606857113 27698142 PMC5081576

[77] Dorighi K.M. Swigut T. Henriques T. Bhanu N.V. Scruggs B.S. Nady N et al 2017 Mll3 and Mll4 Facilitate Enhancer RNA synthesis and transcription from promoters independently of H3K4 Monomethylation Mol. Cell 66 568 576 10.1016/j.molcel.2017.04.018 28483418 PMC5662137

[78] Cao K. Collings C.K. Morgan M.A. Marshall S.A. Rendleman E.J. Ozark P.A. et al 2018 An Mll4/COMPASS-Lsd1 epigenetic axis governs enhancer function and pluripotency transition in embryonic stem cells Sci. Adv. 4 eaap8747 10.1126/sciadv.aap8747 29404406 PMC5796793

[79] Kubo N. Chen P.B. Hu R. Ye Z. Sasaki H. Ren B 2024 H3K4me1 facilitates promoter-enhancer interactions and gene activation during embryonic stem cell differentiation Mol. Cell 84 1742 1752 10.1016/j.molcel.2024.02.030 38513661 PMC11069443

[80] Yan J. Chen S.A.A. Local A. Liu T. Qiu Y. Dorighi K.M. et al 2018 Histone H3 lysine 4 monomethylation modulates long-range chromatin interactions at enhancers Cell Res. 28 204 220 10.1038/cr.2018.1 29313530 PMC5799818

[81] Kumar B. Elsässer S.J 2019 Quantitative multiplexed chip reveals global alterations that shape promoter bivalency in ground state embryonic stem cells Cell Rep 28 3274 3284 10.1016/j.celrep.2019.08.046 31533047 PMC6859498

[82] O’Carroll D. Erhardt S. Pagani M. Barton S.C. Surani M.A. Jenuwein T 2001 The polycomb-group gene Ezh2 is required for early mouse development Mol. Cell. Biol. 21 4330 4336 10.1128/MCB.21.13.4330-4336.2001 11390661 PMC87093

[83] Faust C. Schumacher A. Holdener B. Magnuson T 1995 The eed mutation disrupts anterior mesoderm production in mice Development 121 273 285 10.1242/dev.121.2.273 7768172

[84] Pasini D. Bracken A.P. Jensen M.R. Lazzerini Denchi E. Helin K 2004 Suz12 is essential for mouse development and for EZH2 histone methyltransferase activity EMBO J. 23 4061 4071 10.1038/sj.emboj.7600402 15385962 PMC524339

[85] Schoenfelder S. Sugar R. Dimond A. Javierre B.-M. Armstrong H. Mifsud B. et al 2015 Polycomb repressive complex PRC1 spatially constrains the mouse embryonic stem cell genome Nat. Genet. 47 1179 1186 10.1038/ng.3393 26323060 PMC4847639

[86] Cruz-Molina S. Respuela P. Tebartz C. Kolovos P. Nikolic M. Fueyo R et al 2017 PRC2 Facilitates the regulatory topology required for poised enhancer function during pluripotent stem cell differentiation Cell Stem Cell 20 689 705 10.1016/j.stem.2017.02.004 28285903

[87] Boyle S. Flyamer I.M. Williamson I. Sengupta D. Bickmore W.A. Illingworth RS 2020 A central role for canonical PRC1 in shaping the 3D nuclear landscape Genes Dev. 34 931 949 10.1101/gad.336487.120 32439634 PMC7328521

[88] Grau D. Zhang Y. Lee C.-H. Valencia-Sánchez M. Zhang J. Wang M. et al 2021 Structures of monomeric and dimeric PRC2:EZH1 reveal flexible modules involved in chromatin compaction Nat. Commun. 12 714 10.1038/s41467-020-20775-z 33514705 PMC7846606

[89] Heenan P.R. Wang X. Gooding A.R. Cech T.R. Perkins TT 2020 Bending and looping of long DNA by Polycomb repressive complex 2 revealed by AFM imaging in liquid Nucleic Acids Res. 48 2969 2981 10.1093/nar/gkaa073 32043141 PMC7102998

[90] Mantsoki A. Parussel K. Joshi A 2021 Identification and characterisation of putative enhancer elements in mouse embryonic stem cells Bioinform. Biol. Insights 15 10.1177/1177932220974623 33623376 PMC7876754

[91] Crispatzu G. Rehimi R. Pachano T. Bleckwehl T. Cruz-Molina S. Xiao C. et al 2021 The chromatin, topological and regulatory properties of pluripotency-associated poised enhancers are conserved in vivo Nat. Commun. 12 4344 10.1038/s41467-021-24641-4 34272393 PMC8285398

[92] Leitch H.G. McEwen K.R. Turp A. Encheva V. Carroll T. Grabole N. et al 2013 Naive pluripotency is associated with global DNA hypomethylation Nat. Struct. Mol. Biol. 20 311 316 10.1038/nsmb.2510 23416945 PMC3591483

[93] Richard Albert J. Urli T. Monteagudo-Sánchez A. Le Breton A. Sultanova A. David A. et al 2025 DNA methylation shapes the Polycomb landscape during the exit from naive pluripotency Nat. Struct. Mol. Biol. 32 346 357 10.1038/s41594-024-01405-4 39448850

[94] Jackson M. Krassowska A. Gilbert N. Chevassut T. Forrester L. Ansell J. et al 2004 Severe global DNA hypomethylation blocks differentiation and induces histone hyperacetylation in embryonic stem cells Mol. Cell. Biol. 24 8862 8871 10.1128/MCB.24.20.8862-8871.2004 15456861 PMC517875

[95] Schulz M. Teissandier A. De La Mata Santaella E. Armand M. Iranzo J. El Marjou F. et al 2024 DNA methylation restricts coordinated germline and neural fates in embryonic stem cell differentiation Nat. Struct. Mol. Biol. 31 102 114 10.1038/s41594-023-01162-w 38177678

[96] Argelaguet R. Clark S.J. Mohammed H. Stapel L.C. Krueger C. Kapourani C.-A. et al 2019 Multi-omics profiling of mouse gastrulation at single-cell resolution Nature 576 487 491 10.1038/s41586-019-1825-8 31827285 PMC6924995

[97] Dawlaty M.M. Breiling A. Le T. Barrasa M.I. Raddatz G. Gao Q et al 2014 Loss of Tet enzymes compromises proper differentiation of embryonic stem cells Dev. Cell 29 102 111 10.1016/j.devcel.2014.03.003 24735881 PMC4035811

[98] Yin Y. Morgunova E. Jolma A. Kaasinen E. Sahu B. Khund-Sayeed S. et al 2017 Impact of cytosine methylation on DNA binding specificities of human transcription factors Science 356 eaaj2239 10.1126/science.aaj2239 28473536 PMC8009048

[99] Kreibich E. Kleinendorst R. Barzaghi G. Kaspar S. Krebs A.R 2023 Single-molecule footprinting identifies context-dependent regulation of enhancers by DNA methylation Mol. Cell 83 787 802 10.1016/j.molcel.2023.01.017 36758546

[100] Davidson I.F. Peters JM 2021 Genome folding through loop extrusion by SMC complexes Nat. Rev. Mol. Cell Biol. 22 445 464 10.1038/s41580-021-00349-7 33767413

[101] Liu N.Q. Maresca M. van den Brand T Braccioli L. Schijns M.M.G.A. Teunissen H. et al 2021 WAPL maintains a cohesin loading cycle to preserve cell-type-specific distal gene regulation Nat. Genet. 53 100 109 10.1038/s41588-020-00744-4 33318687 PMC7610352

[102] Bird A 2025 Cohesin as an essential disruptor of chromosome organization Mol. Cell 85 1054 1057 10.1016/j.molcel.2025.01.010 39909042 PMC7619274

[103] Ramasamy S. Aljahani A. Karpinska M.A. Cao T.B.N. Velychko T. Cruz J.N. et al 2023 The Mediator complex regulates enhancer-promoter interactions Nat. Struct. Mol. Biol. 30 991 1000 10.1038/s41594-023-01027-2 37430065 PMC10352134

[104] Kane L. Williamson I. Flyamer I.M. Kumar Y. Hill R.E. Lettice L.A. et al 2022 Cohesin is required for long-range enhancer action at the Shh locus Nat. Struct. Mol. Biol. 29 891 897 10.1038/s41594-022-00821-8 36097291 PMC7613721

[105] Hsieh T.H.S. Cattoglio C. Slobodyanyuk E. Hansen A.S. Darzacq X. Tjian R 2022 Enhancer–promoter interactions and transcription are largely maintained upon acute loss of CTCF, cohesin, WAPL or YY1 Nat. Genet. 54 1919 1932 10.1038/s41588-022-01223-8 36471071 PMC9729117

[106] Rao S.S.P. Huang S.-C. Glenn St Hilaire B. Engreitz J.M. Perez E.M. Kieffer-Kwon K.-R et al 2017 Cohesin loss eliminates all loop domains Cell 171 305 320 10.1016/j.cell.2017.09.026 28985562 PMC5846482

[107] Wutz G. Várnai C. Nagasaka K. Cisneros D.A. Stocsits R.R. Tang W. et al 2017 Topologically associating domains and chromatin loops depend on cohesin and are regulated by CTCF, WAPL, and PDS5 proteins EMBO J. 36 3573 3599 10.15252/embj.201798004 29217591 PMC5730888

[108] Hansen K.L. Adachi A.S. Braccioli L. Kadvani S. Boileau R.M. Pokorny B. et al 2024 Synergy between cis -regulatory elements can render cohesin dispensable for distal enhancer function Molecular Biology 2024 Molecular Biology 10.1101/2024.10.04.615095 PMC13446962 41308125

[109] Lettice L.A. Heaney S.J.H. Purdie L.A. Li L. de Beer P. Oostra B.A. et al 2003 A long-range Shh enhancer regulates expression in the developing limb and fin and is associated with preaxial polydactyly Hum. Mol. Genet. 12 1725 1735 10.1093/hmg/ddg180 12837695

[110] Lettice L.A. Devenney P. De Angelis C. Hill R.E 2017 The conserved sonic hedgehog limb enhancer consists of discrete functional elements that regulate precise spatial expression Cell Rep. 20 1396 1408 10.1016/j.celrep.2017.07.037 28793263 PMC5561167

[111] Williamson I. Lettice L.A. Hill R.E. Bickmore WA 2016 Shh and ZRS enhancer colocalisation is specific to the zone of polarising activity Development 143 2994 3001 10.1242/dev.139188 27402708 PMC5004883

[112] Benabdallah N.S. Williamson I. Illingworth R.S. Kane L. Boyle S. Sengupta D et al 2019 Decreased enhancer-promoter proximity accompanying enhancer activation Mol. Cell 76 473 484 10.1016/j.molcel.2019.07.038 31494034 PMC6838673

[113] Alexander J.M. Guan J. Li B. Maliskova L. Song M. Shen Y. et al . Live-cell imaging reveals enhancer-dependent Sox2 transcription in the absence of enhancer proximity Elife 8 10.7554/eLife.41769 PMC6534382 31124784

[114] Gassler J. Brandão H.B. Imakaev M. Flyamer I.M. Ladstätter S. Bickmore W.A. et al 2017 A mechanism of cohesin-dependent loop extrusion organizes zygotic genome architecture EMBO J. 36 3600 3618 10.15252/embj.201798083 29217590 PMC5730859

[115] Stolper R.J. Tsang F.H. Georgiades E. Hansen L.L.P. Downes D.J. Harrold C.L et al 2023 Loop extrusion by cohesin plays a key role in enhancer-activated gene expression during differentiation Mol Biol (Los Angel) 09.07.556660 10.1101/2023.09.07.556660 PMC13402345 42192110

[116] Goel V.Y. Huseyin M.K. Hansen AS 2023 Region Capture Micro-C reveals coalescence of enhancers and promoters into nested microcompartments Nat. Genet. 55 1048 1056 10.1038/s41588-023-01391-1 37157000 PMC10424778

[117] Thomas H.F. Feng S. Haslhofer F. Huber M. García Gallardo M. Loubiere V et al 2025 Enhancer cooperativity can compensate for loss of activity over large genomic distances Mol. Cell 85 362 375 10.1016/j.molcel.2024.11.008 39626663

[118] Xie L. Torigoe S.E. Xiao J. Mai D.H. Li L. Davis F.P. et al 2017 A dynamic interplay of enhancer elements regulates *Klf4* expression in naïve pluripotency Genes Dev. 31 1795 1808 10.1101/gad.303321.117 28982762 PMC5666677

[119] Amblard I. Baranasic D. Xie S.Q. Moyon B. Percharde M. Lenhard B et al 2025 A dual enhancer-attenuator element ensures transient Cdx2 expression during mouse posterior body formation Dev. Cell S1534-5807 00361 00362 10.1016/j.devcel.2025.06.006 40580963 PMC12979250

[120] Lalanne J.-B. Regalado S.G. Domcke S. Calderon D. Martin B.K. Li X. et al 2024 Multiplex profiling of developmental cis-regulatory elements with quantitative single-cell expression reporters Nat. Methods 21 983 993 10.1038/s41592-024-02260-3 38724692 PMC11166576

[121] Acampora D. Di Giovannantonio L.G. Simeone A 2013 Otx2 is an intrinsic determinant of the embryonic stem cell state and is required for transition to a stable epiblast stem cell condition Development 140 43 55 10.1242/dev.085290 23154415

[122] Matsuo I. Kuratani S. Kimura C. Takeda N. Aizawa S 1995 Mouse Otx2 functions in the formation and patterning of rostral head Genes Dev. 9 2646 2658 10.1101/gad.9.21.2646 7590242

[123] King D.M. Hong C.K.Y. Shepherdson J.L. Granas D.M. Maricque B.B. Cohen B.A . Synthetic and genomic regulatory elements reveal aspects of cis-regulatory grammar in mouse embryonic stem cells Elife 9 10.7554/eLife.41279 PMC7077988 32043966

[124] Leeb M. Dietmann S. Paramor M. Niwa H. Smith A 2014 Genetic exploration of the exit from self-renewal using haploid embryonic stem cells Cell Stem Cell 14 385 393 10.1016/j.stem.2013.12.008 24412312 PMC3995090

[125] Yi F. Pereira L. Hoffman J.A. Shy B.R. Yuen C.M. Liu D.R. et al 2011 Opposing effects of Tcf3 and Tcf1 control Wnt stimulation of embryonic stem cell self-renewal Nat. Cell Biol. 13 762 770 10.1038/ncb2283 21685894 PMC3129424

[126] Wray J. Kalkan T. Gomez-Lopez S. Eckardt D. Cook A. Kemler R. et al 2011 Inhibition of glycogen synthase kinase-3 alleviates Tcf3 repression of the pluripotency network and increases embryonic stem cell resistance to differentiation Nat. Cell Biol. 13 838 845 10.1038/ncb2267 21685889 PMC3160487

[127] Kalkan T. Bornelöv S. Mulas C. Diamanti E. Lohoff T. Ralser M. et al 2019 Complementary activity of ETV5, RBPJ, and TCF3 drives formative transition from naive pluripotency Cell Stem Cell 24 785 801 10.1016/j.stem.2019.03.017 31031137 PMC6509416

